# Anatomical Charts

**Published:** 1881-06

**Authors:** 


### EDITORIAL.

### ANATOMICAL CHARTS.

As an aid to the Student of Anatomy in the prosecution of his studies, and also to spare him the bewilderment caused by wading through the mass of verbiage with which our text books are encumbered, for facts which could and should be briefly stated. Prof. C. L. Ford, of the Medical department of the University of Michigan, has prepared and published two Charts. One containing a classification of the Muscles of the human body with their origin, insertion, nervous supply and principal action of each. These are arranged in six different columns. The first giving the name of the group to which the muscle belongs; the second, the origin, etc. This arrangement enables the student at a glance to obtain the information he desires. The other is a classification of the Cranial Nerves, in which they are arranged in twelve pairs instead of nine, as is usually done; giving the name, origin, foramen of exit, principal distribution and function—these are also placed in columns, thus simplifying their study to a great degree. Charts such as these would be valuable in the dissecting room, saving much of the student's time, and enabling him to devote more attention to actual observation, which after all, is the only way to acquire a thorough anatomical knowledge—the only knowledge that is of any value, is that which he gains by observation when thrown upon his own resources.

